# Connecting virulence pathways to cell-cycle progression in the fungal pathogen *Cryptococcus neoformans*

**DOI:** 10.1007/s00294-017-0688-5

**Published:** 2017-03-06

**Authors:** Christina M. Kelliher, Steven B. Haase

**Affiliations:** Department of Biology, Duke University, Box 90338, 130 Science Drive, Durham, NC 27708-0338 USA

**Keywords:** *Cryptococcus neoformans*, Cell-cycle transcription, Virulence factors, Gene regulatory networks

## Abstract

Proliferation and host evasion are critical processes to understand at a basic biological level for improving infectious disease treatment options. The human fungal pathogen *Cryptococcus neoformans* causes fungal meningitis in immunocompromised individuals by proliferating in cerebrospinal fluid. Current antifungal drugs target “virulence factors” for disease, such as components of the cell wall and polysaccharide capsule in *C. neoformans*. However, mechanistic links between virulence pathways and the cell cycle are not as well studied. Recently, cell-cycle synchronized *C. neoformans* cells were profiled over time to identify gene expression dynamics (Kelliher et al., PLoS Genet 12(12):e1006453, 2016). Almost 20% of all genes in the *C. neoformans* genome were periodically activated during the cell cycle in rich media, including 40 genes that have previously been implicated in virulence pathways. Here, we review important findings about cell-cycle-regulated genes in *C. neoformans* and provide two examples of virulence pathways—chitin synthesis and G-protein coupled receptor signaling—with their putative connections to cell division. We propose that a “comparative functional genomics” approach, leveraging gene expression timing during the cell cycle, orthology to genes in other fungal species, and previous experimental findings, can lead to mechanistic hypotheses connecting the cell cycle to fungal virulence.

## Introduction

Human fungal pathogens cause more than a million life-threatening illnesses each year (Brown et al. 2012). Antifungal drug development focuses on targeting the pathogen without causing significant side effects in the host. The cell cycle is highly conserved across eukaryotic species, because it is an essential process for growth and division. Thus, cell-cycle machinery is not an ideal candidate for antifungal drug design. However, connections between the cell cycle and fungal-specific virulence factors are poorly understood. An improved basic biological understanding of fungal proliferation and links to virulence pathways can increase drug treatment options.

The cell division cycle is a fundamental biological process underlying growth and reproduction. The cell cycle is divided into four phases (Gap 1, Synthesis, Gap 2, and Mitosis), where cells precisely duplicate their genomic content and then faithfully segregate cellular contents into two new cells (Morgan 2007). These cell-cycle events, such as DNA replication and spindle formation, are regulated by cyclin-dependent kinases (CDKs) and their cyclin-binding partners (Bloom and Cross 2007; Evans et al. 1983; Hartwell et al. 1974; Nasmyth 1993; Nurse and Thuriaux 1980). In addition to driving periodic cellular events, many genes encoding cell-cycle regulators are themselves periodically transcribed. Programs of periodic gene expression have been observed in many eukaryotes including fungi, plants, mice fibroblasts, and human cell lines (Bar-Joseph et al. 2008; Grant et al. 2013; Ishida et al. 2001; Menges et al. 2005; Oliva et al. 2005; Peng et al. 2005; Rustici et al. 2004). In the budding yeast *Saccharomyces cerevisiae*, transcription of periodic genes in the proper cell-cycle phase is controlled by transcription factors, which are also regulated at the protein level by cyclin/CDKs and by ubiquitin ligases such as the APC/C (Bristow et al. 2014; Landry et al. 2014; Lee et al. 2002; Orlando et al. 2008; Ostapenko and Solomon 2011; Simmons Kovacs et al. 2012; Simon et al. 2001) (reviewed in: Benanti 2016; Haase and Wittenberg 2014).

*Cryptococcus neoformans* (Basidiomycota) is a distantly related budding yeast to *S. cerevisiae* (Ascomycota) (Stajich et al. 2009), but the cell cycle is not as well characterized in C. *neoformans. C. neoformans* can cause a respiratory infection with pneumonia-like symptoms in the lungs, followed by dissemination and proliferation in the human central nervous system. Fungal meningitis and other infections are a leading cause of death in immune-compromised individuals (Brown et al. 2012; Park et al. 2009). The most effective antifungal treatments for cryptococcosis target *C. neoformans* cells without affecting host cells. Therefore, the translational realm of the *C. neoformans* field studies “virulence factors” for fungal disease, such as the yeasts’ cell wall and polysaccharide capsule (O’Meara and Alspaugh 2012). The cell cycle has not traditionally been considered a virulence factor, but many virulence functions appear to be under cell-cycle control, so mechanisms controlling cell division will impact fungal virulence. For example, one G1 cyclin gene has been identified in *C. neoformans* (CNAG_06092), and its mRNA is expressed periodically during the cell cycle (Kelliher et al. 2016). The *CLN1* gene is not essential for viability, but the knockout strain is defective in proliferation at 37 °C, less virulent in an insect model, grows to abnormally large cell sizes, lacks melanin production, and shows polysaccharide capsule defects compared to wild-type controls (García-Rodas et al. 2014, 2015). These genetic findings directly connect cell-cycle machinery defects to canonical virulence pathways. Interestingly, a recent screen for essential genes *C. neoformans* identified ribosomal RNA and other metabolic regulators but did not identify any putative cell-cycle genes (Kuwada et al. 2015). Thus, there is still much to be learned about the cell cycle in *C. neoformans*, as the only known G1 cyclin gene and other putative key regulators of the cell cycle do not appear to be required for viability.

In this review, we highlight the importance of combining transcriptome dynamics with functional studies. We investigate two virulence pathways in *C. neoformans* that contain genes that are periodically expressed during the cell cycle. We find that multiple enzymes controlling chitin synthesis are co-expressed periodically in a specific cell-cycle phase. On the other hand, genes involved in mating pheromone sensing are expressed in different cell-cycle phases. After identifying the expression timing of the virulence genes of interest, we predict roles of these genes during the cell cycle by incorporating the previous genetic and cell biological findings about gene function and by comparing to sequence orthologs in *S. cerevisiae*. These two virulence pathways serve as examples for the types of mechanistic hypotheses that can be generated from understanding the gene expression dynamics of the *C. neoformans* cell cycle. We close with a discussion of the direction of this functional genomics work—constructing gene regulatory networks that explain how large programs of periodic genes are controlled during the fungal cell cycle.

## Phase-specific gene expression during the fungal cell cycle

**Fig. 1:**
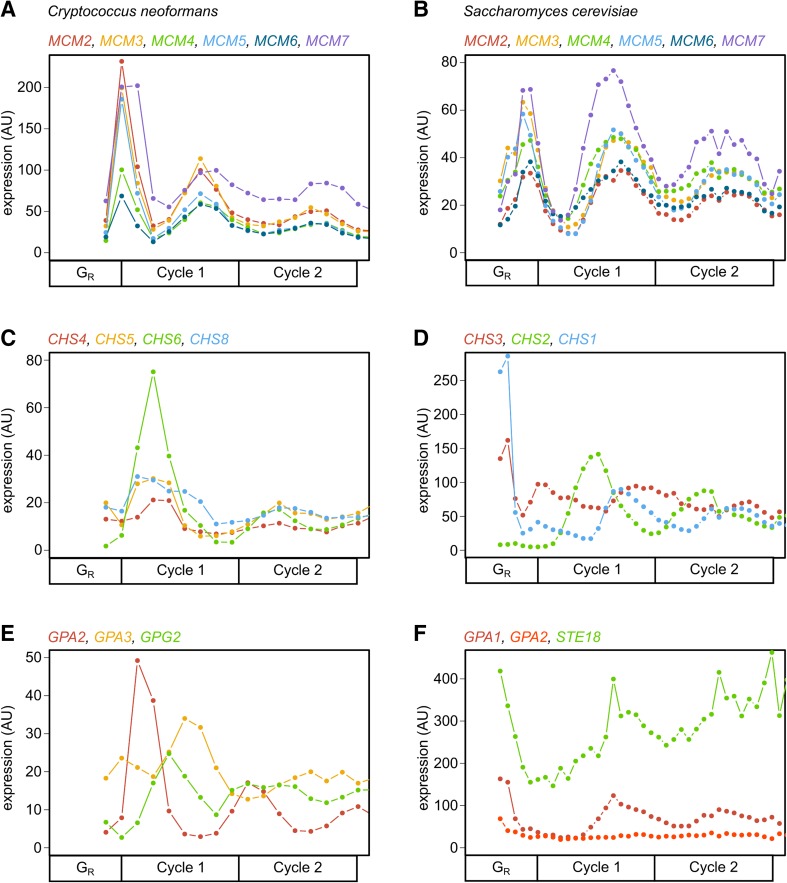
Timing of expression provides mechanistic insights for DNA replication, chitin synthase, and GPCR subunits during the *C. neoformans* and *S. cerevisiae* cell cycles. The *MCM2-7* genes involved in DNA replication origin firing are plotted in *C. neoformans* (respectively: CNAG_03341, CNAG_00099, CNAG_06182, CNAG_04052, CNAG_03962, and CNAG_05825) (**a**) and *S. cerevisiae* (respectively: YBL023C, YEL032W, YPR019W, YLR274W, YGL201C, and YBR202W) (**b**) to visualize activation timing before S phase of the cell cycle. Chitin synthase genes in *C. neoformans* are expressed after S phase (**c**), while *S. cerevisiae* orthologs vary in their expression timing (**d**). *C.n. CHS4* is orthologous to *S.c. CHS3* (YBR023C, *red* **c, d**). Both *CHS6* and *CHS8* have orthology to *CHS1* (YNL192W) and *CHS2* (YBR038W) in *S. cerevisiae*. According to a global sequence similarity measure (Kelliher et al. 2016: S4 Table), *C.n. CHS6* is most similar to *S.c. CHS2* (*green* **c, d**), and *C.n. CHS8* is more similar to *S.c. CHS1* (*blue* **c, d**). GPCR subunits in *C. neoformans* are expressed at different times during the cell cycle (**e**), and *S. cerevisiae* orthologs are less periodic and vary in expression timing (**f**). *C.n. GPA2* is orthologous to both *S.c. GPA1* (YHR005C) and *GPA2* (YER020W, *red lines* **e, f**), and *GPG2* is orthologous to *STE18* (YJR086W) in *S. cerevisiae*. In all plots, orthologous gene pairs are shown in the *same color*, and ortholog identification data can be found in the previous work (Kelliher et al. 2016: S4 Table, S1 File). Line plots are shown on an fpkm unit scale, which were normalized separately for each yeast experiment. All transcripts are plotted on a common cell-cycle timeline in CLOCCS lifeline points as described (Kelliher et al. 2016: S1 File). Periodicity rankings for each *C. neoformans* gene can be found in S2 Table, and *S. cerevisiae* genes can be found in S1 Table (Kelliher et al. 2016)

Periodic cell-cycle genes have been characterized extensively in the budding yeast *S. cerevisiae* (Bristow et al. 2014; Cho et al. 1998; de Lichtenberg et al. 2005; Eser et al. 2014; Granovskaia et al. 2010; Hereford et al. 1981; Orlando et al. 2008; Pramila et al. 2006; Spellman et al. 1998). In *S. cerevisiae*, many cell-cycle genes peak in mRNA expression level before their protein products are used in cell cycle events. One canonical example is DNA replication origin firing, where replication origin proteins are assembled into a complex before S phase, activated, and then degraded or changed in localization to prevent re-replication (Bell and Dutta 2002). This “just-in-time transcription” phenomenon can be visualized for the conserved DNA helicase complex that acts during origin firing in both *S. cerevisiae* and *C. neoformans* (Fig. 1a, b). Our recent publication describes in detail how different time series experiments for *C. neoformans* and *S. cerevisiae* cells are aligned on a common “cell-cycle time” axis using the CLOCCS algorithm (Orlando et al. 2007, 2009) (Kelliher et al. 2016: S1 File). According to this common timeline, origin-firing genes are transcribed in early G1 phase during each cell cycle (Guo et al. 2013; Kelliher et al. 2016). These findings suggest a common function for *MCM* genes in *S. cerevisiae* and in *C. neoformans*.

Comparative genomics was also applied to groups of co-regulated cell-cycle genes (Kelliher et al. 2016). We found that DNA replication (S phase) and mitosis (M phase) genes in *S. cerevisiae* and *C. neoformans* were highly conserved in periodicity and timing of expression during the fungal cell cycle. These analyses required identification of orthologous genes in the two species of budding yeast, and our recent publication describes in detail how orthologous genes were identified in *C. neoformans* and *S. cerevisiae* (Kelliher et al. 2016: S1 File). Almost one thousand additional periodic genes were identified in *C. neoformans*, many of which have not been previously linked to the cell cycle. We posit that identifying the phase in which these unknown genes are expressed can provide mechanistic insights. For example, *S. cerevisiae* genes that play a role in bud emergence peak in expression before G1/S phase. Orthologous genes in *C. neoformans* were not highly conserved in periodicity or timing of expression at G1/S phase (Kelliher et al. 2016: Figure 4). This putative divergence in budding gene timing is supported by data that *C. neoformans* bud emergence can occur in a range of times between G1 and G2 phases, depending on culturing conditions such as oxygen levels and cell concentration (Ohkusu et al. 2001, 2004). Thus, gene orthology alone is not necessarily informative regarding biological function across fungal systems. Below, we investigate gene expression timing of two virulence pathways to connect virulence mechanisms to cell-cycle progression.

## Chitin synthesis in *C. neoformans* may be directly linked to cell-cycle progression

Genes involved in virulence pathways are of critical importance for understanding the biology and for treating the opportunistic fungal pathogen *C. neoformans* (Buchanan and Murphy 1998; Liu et al. 2008). Our recent publication identified 40 periodic genes that have been previously identified by genetic screens for virulence phenotypes (Kelliher et al. 2016: S3 Table). Here, we asked if any metabolic pathways were enriched in this list of 40 virulence genes using the database FungiDB (Stajich et al. 2012). The most significant Metabolic Pathway hit (PWY-6981) included four genes involved in chitin biosynthesis. These four chitin synthase enzymes—*CHS4* (CNAG_00546), *CHS5* (CNAG_05818), *CHS6* (CNAG_06487), and *CHS8* (CNAG_07499)—are periodically expressed during the *C. neoformans* cell cycle (Kelliher et al. 2016).

Chitin synthesis is a ubiquitous and dynamic process across fungal species (Langner and Göhre 2016). The previous work has characterized the family of chitin synthase genes and shown that chitin and chitosan levels accumulate along with population density in *C. neoformans*, unlike the budding yeasts *S. cerevisiae* and *Candida albicans* (Banks et al. 2005). The *CHS3* gene is highly expressed in proliferating *C. neoformans* cells, and single *chs3* mutants are temperature sensitive at 37 °C, which is a highly relevant virulence factor for human infection (Bloom and Panepinto 2014). In addition to the previous work on steady-state expression levels of chitin synthase genes from asynchronous *C. neoformans* cells (Banks et al. 2005: Figure 3), the cell-cycle time series data set now provides much more dynamical detail. We visualized the periodic chitin synthase genes to determine their timing of peak expression during the cell cycle.

The four periodic chitin synthase genes are co-expressed and peak in expression after the S phase *MCM* genes in *C. neoformans* (Fig. 1a, c). The previous work showed that chitin/chitosan levels in the cell wall vary between *S. cerevisiae* and *C. neoformans* (Banks et al. 2005), and thus, it was important to compare these chitin genes to their putative orthologous genes in *S. cerevisiae*. In *C. neoformans*, chitin synthase genes are much more coordinately expressed in time than their *S. cerevisiae* orthologs (Fig. 1c, d). The *S. cerevisiae* gene *CHS2* is most similar in dynamics and expression timing to the group of *C. neoformans* chitin synthases. The *S. cerevisiae CHS2* gene plays a role in cell wall remodeling during cytokinesis (Oh et al. 2012; Sburlati and Cabib 1986), while *CHS1* and *CHS3* affect chitin levels in the *S. cerevisiae* cell wall during alpha-factor arrest (shmoo formation), during bud emergence, and generally during cell growth (Shaw et al. 1991).

We hypothesize that the four periodically expressed chitin synthase enzymes in *C. neoformans* are utilized after S phase for bud growth and/or during cytokinesis for extracellular matrix remodeling. Unlike *S. cerevisiae*, the expression of *CHS4, CHS5, CHS6*, and *CHS8* is tightly coordinated, suggesting they act at the same time to perform a similar function. The transcription factor(s) controlling the coordinated activation of *CHS* genes is unknown. The *CRZ1* transcription factor (CNAG_00156) is known to regulate *CHS6* expression levels under various stress conditions (Lev et al. 2012), but the *CRZ1* transcript did not score highly for cell-cycle periodicity (Kelliher et al. 2016: S2 Table). If the transcriptional regulator(s) can be identified, a combination drug therapy (Bahn 2015; Zhang et al. 2014) to stall the fungal cell cycle in G2 or M phase and simultaneously inhibit chitin synthase could render cells as poorly virulent as *chs3* mutants in the laboratory (Banks et al. 2005). Chitin synthesis represents a promising antifungal target for further study.

## A subset of G-protein coupled receptor subunits are expressed at different times during the *C. neoformans* cell cycle

Given the 40 periodic genes with previously identified virulence phenotypes (Kelliher et al. 2016: S3 Table), we also used FungiDB to ask if any Gene Ontology terms were enriched (Stajich et al. 2012). G-protein coupled receptor signaling (GO:0007186) was one of the top five most significant GO terms. G-protein coupled receptor (GPCR) signaling pathways have been studied extensively in *C. neoformans* for their role in sensing and responding to the cellular environment (Xue et al. 2008). The three periodically expressed GPCR subunits have previously been implicated in the signaling pathway that allows haploid *C. neoformans* cells to sense the opposite mating type via mating pheromones. During infection, *C. neoformans* cells are typically haploid and proliferating asexually. However, meiotic spores are thought to initiate the first steps of host colonization in the lungs, and thus, understanding the biology of both the asexual and sexual phases of *C. neoformans* growth is essential (Kozubowski and Heitman 2012).

Three periodic GPCR subunits are expressed at different times during the cell cycle (Fig. 1e). *GPG2* (CNAG_05890) is a Gγ subunit, which can bind to Gβ subunits in two different signaling pathways: Gib2 (CNAG_05465), associated with nutrient sensing, and Gpb1 (CNAG_05465), associated with mating pheromone sensing (Palmer et al. 2006). *GPA2* (CNAG_00179) and *GPA3* (CNAG_02090) are Gα subunits, and are expressed in different phases of the cell cycle (Fig. 1e). The previous work has shown that *GPA2* activates mating, while *GPA3* inhibits mating, but both Gα genes must be deleted for a fungal sterility phenotype (Hsueh et al. 2007). The mating pathway in *S. cerevisiae* is well understood and has fewer components than *C. neoformans* (Bardwell 2004; Dohlman and Thorner 2001). In *S. cerevisiae*, the G-protein subunits involved in mating pheromone signaling are *GPA1* (Gα), *STE18* (Gγ), and *STE4* (Gβ). The *S. cerevisiae GPA1* subunit is periodically expressed during the cell cycle, but its peak expression timing does not precisely match its ortholog in *C. neoformans* (Fig. 1e, f).

Unlike *GPA1* in *S. cerevisiae*, the Gα subunits of the *C. neoformans* mating pathway (*GPA2* and *GPA3*) peak in different phases of the cell cycle. Perhaps, *C. neoformans* cells are capable of sensing mating pheromone throughout the cell cycle, rather than exclusively G1 phase. Alternatively, these GPCRs may have been repurposed for other functions in *C. neoformans*. The strong peak of *GPA2* expression at each G1 phase in *C. neoformans* does suggest that, like *S. cerevisiae*, cells may be “deciding” whether or not to mate before commitment to each cell cycle (Fig. 1e, f). Intriguingly, mating in *C. neoformans* is also linked to light–dark cycles and regulated by the circadian rhythm transcription factor orthologs *BWC1* (CNAG_05181) and *BWC2* (CNAG_02435) (Idnurm and Heitman 2005). These two TF genes do not score as highly periodic during the *C. neoformans* cell cycle (Kelliher et al. 2016: S2 Table), but the contribution of circadian rhythms to virulence is not well understood for many pathogenic species and warrants further study (Hevia et al. 2016).

## Future directions: building gene regulatory networks

In this review, we highlight what can be learned about gene function by the pattern of expression throughout the cell cycle. By combining information about expression dynamics with orthology and functional studies from model systems, we demonstrate that new mechanistic hypotheses can be rapidly generated. Here, we begin to elucidate connections between the cell cycle and virulence pathways using these approaches. We show that four chitin synthases in *C. neoformans* are co-expressed after S phase, unlike their putative orthologs in *S. cerevisiae* (Fig. 1c, d). We also demonstrate that two Gα subunits involved in mating pheromone signaling are expressed in different cell-cycle phases, where their putative *S. cerevisiae* ortholog is expressed only prior to G1 phase (Fig. 1e, f). By combining information from timing of expression during the cell cycle, evolutionarily related genes, and previous functional work in a “comparative functional genomics” approach, we can build mechanistic, testable hypotheses about virulence gene function in non-model organisms. Both *C. neoformans* and *S. cerevisiae* budding yeasts provide supporting evidence for the “just-in-time transcription” hypothesis, where sets of genes are co-expressed at a given time to perform a function during a specific cell-cycle phase (Kelliher et al. 2016). In future work, the approaches described here can be applied to the many additional periodic genes in *C. neoformans* with unknown cell-cycle functions.

A long-term goal of this work is to characterize the regulatory pathways that control periodic gene expression during the fungal cell cycle. Identification of transcription factors and of their binding sites in the genome will be essential knowledge to approach this problem. These data sets are available in *S. cerevisiae*, which quantitative models have used to predict an interconnected network of periodically expressed transcription factors that are capable of driving cell-cycle transcription (Hillenbrand et al. 2016; Li et al. 2004; Orlando et al. 2008; Sevim et al. 2010; Simmons Kovacs et al. 2012). Transcription factor (TF) deletion collections have been generated and carefully phenotyped in both *C. neoformans* (Jung et al. 2015) and *C. albicans* (Homann et al. 2009). One promising avenue of future work would be to synchronize mutant cells in the cell cycle and determine if single or double TF mutants affect cell-cycle progression (Simmons Kovacs et al. 2012).

One historical example of a characterized gene regulatory network that regulates periodic gene expression is the circadian rhythm, which is present in almost all organisms to anticipate environmental light–dark cycles (Zhang and Kay 2010). Using *Neurospora crassa* as a model for the eukaryotic circadian clock, mutations in the *frequency* (*FRQ*) locus were identified in screens for arrhythmic fungi (Loros and Feldman 1986; McClung et al. 1989). The FRQ protein participates in a core negative feedback loop that regulates the circadian period length (Hurley et al. 2016). The screens that identified *FRQ* in *N. crassa* and its orthologs in other eukaryotes represent the utility of genetic approaches when one gene has a large effect on the biological phenotype of interest (analogous to mapping human disease genes with Mendelian inheritance).

Complex phenotypes and quantitative traits are more challenging to solve using genetic approaches alone. For example, the positive regulators of the *N. crassa* circadian network, white collar-1 (*WC-1*) and white collar-2 (*WC-2*), were characterized more than a decade later than *FRQ* due to complex and partially redundant roles in activating light-responsive genes in the network (Crosthwaite et al. 1997). Redundancy in biological processes can be addressed with double mutant screens that are more effective at identifying core genes controlling a dynamic process (Costanzo et al. 2013). However, robustness can come from multiple genes with partially overlapping functions or, in response to genetic perturbation, from “compensation” in the strength of interactions in a network transcription factors, which has directly been demonstrated in the mammalian circadian clock network (Baggs et al. 2009). Intricate network interactions provide robustness to the network, but can foil the traditional genetic approaches, and thus network modeling of dynamical systems will become an invaluable approach for learning complex mechanisms.

We posit that cell-cycle networks, like the circadian rhythm, are also ancient in origin and contain highly redundant regulatory pathways (Simmons Kovacs et al. 2008). In the recent *C. neoformans* publication, the cell-cycle network topology at G1/S phase was highlighted as a region of partial conservation between fungal species (Kelliher et al. 2016: Figure 6). In fact, the transcriptional machinery involved in the cellular “decision” to commit to the cell cycle, enter quiescence, or select another fate is functionally conserved in G1/S phase from *S. cerevisiae* to human cells (Miles and Breeden 2016). Further work is needed to understand interacting genes in pathogenic fungi and also in the conservation of these fungal and animal gene networks (Brown and Madhani 2012; Medina et al. 2016). In addition to quantifying mRNA abundance, the localization and protein activity of TFs and other cell-cycle regulators will be important directions for future work in *C. neoformans* (Chong et al. 2015; Kuwada et al. 2015).

Building networks of genes that control a given process is critical for a full understanding of the dynamics of a biological system. Understanding dynamics and network topology also allows us to predict how gene networks will respond to perturbation (such as drug treatment) and combat drug resistance, which is a major problem in infectious diseases. Preliminary networks of interacting genes have been established in the wheat pathogen *Fusarium graminearum* (Guo et al. 2016), in nitrogen utilization in the pathogenic yeast *C. albicans* (Ramachandra et al. 2014), and during ordered capsule assembly in *C. neoformans* (Maier et al. 2015). We propose that synchronizing populations of cells for the cell cycle could build on this pioneering work and elucidate direct connections between proliferation and virulence factors. Another useful direction for future work on understanding gene regulatory networks in *C. neoformans* will be to profile cell-cycle synchronized cells in non-rich media and/or at high temperature, as it is already known that steady-state gene expression levels change in response to poor media conditions (Janbon et al. 2014).

The opportunistic fungal pathogen *C. neoformans* expresses nearly 20% of all genes periodically during the cell cycle (Kelliher et al. 2016), and we have begun to make connections between virulence pathways and the cell cycle. To elucidate the network of transcription factors and other cell-cycle regulators that control periodic virulence pathways, future work will assay cell-cycle phenotypes from the *C. neoformans* TF deletion collection (Jung et al. 2015) in rich media and in poor media conditions that mimic infection (Janbon et al. 2014). An improved understanding of cell-cycle biology in fungal species will lead to more informed, and potentially combination therapies to treat fungal diseases.
